# Progress in the study of biomarkers for early prediction of systemic inflammatory response syndrome after percutaneous nephrolithotomy

**DOI:** 10.3389/fimmu.2023.1142346

**Published:** 2023-03-30

**Authors:** Wangjian Wu, Di Zhang, Tongtong Jin, Tianyi Lu, Fenghai Zhou

**Affiliations:** ^1^ The First Clinical Medical College of Lanzhou University, Lanzhou, China; ^2^ The First Clinical Medical College of Gansu University of Chinese Medicine (Gansu Provincial Hospital), Lanzhou, China; ^3^ Department of Urology, Gansu Provincial Hospital, Lanzhou, China

**Keywords:** percutaneous nephrolithotripsy, systemic inflammatory response syndrome, biomarkers, PCNL, SIRS

## Abstract

Urolithiasis is a common and frequent disease in urology. Percutaneous nephrolithotomy (PCNL) is preferred for the treatment of upper urinary tract stones and complicated renal stones >2 cm in diameter, but it has a higher rate of postoperative complications, especially infection, compared with other minimally invasive treatments for urinary stones. Complications associated with infection after percutaneous nephrolithotomy include transient fever, systemic inflammatory response syndrome (SIRS), and sepsis, which is considered one of the most common causes of perioperative death after percutaneous nephrolithotomy. In contrast, SIRS serves as a sentinel for sepsis, so early intervention of SIRS by biomarker identification can reduce the incidence of postoperative sepsis, which in turn reduces the length of stay and hospital costs for patients. In this paper, we summarize traditional inflammatory indicators, novel inflammatory indicators, composite inflammatory indicators and other biomarkers for early identification of systemic inflammatory response syndrome after percutaneous nephrolithotomy.

## Introduction

Urolithiasis is a common and frequent disease in urology, and with the change of diet and lifestyle, the incidence of stones has been increasing in the past three decades at home and abroad (1, 2), and the incidence of stones is 8.8% in the United States and about 5.8% in China (3, 4). Urinary stones often lead to colic, infection, decreased kidney function, and even kidney failure, which greatly reduces patients’ quality of life and increases their financial burden. The treatment of urinary stones has evolved from traditional open surgery to minimally invasive endoluminal urological procedures, of which percutaneous nephrolithotomy (PCNL) has become the treatment of choice for patients with upper urinary tract stones >2 cm and complicated renal stones (5). Compared with traditional open surgery, percutaneous nephrolithoscopy has the advantages of minimal invasiveness, short operative time, short hospital stay, and high stone removal rate (6), but the complexity of its own operation and long learning curve make its postoperative complication rate higher, such as postoperative hemorrhage and postoperative infection (7, 8). Complications related to infection after percutaneous nephrolithotomy can be classified as transient fever, systemic inflammatory response syndrome (SIRS), and sepsis, depending on the severity.

Systemic inflammatory response syndrome is an uncontrolled, self-destructive, and self-sustained amplified systemic inflammatory response caused by severe injury, infection, trauma, surgery, ischemia, and other factors (9), and is also one of the common complications after percutaneous nephrolithotomy, with a high incidence even with preoperative antibiotic prophylaxis. The incidence of systemic inflammatory response syndrome after percutaneous nephrolithotomy has been reported to be approximately 9.8%-43% (10). In addition, the presence of SIRS during hospitalization is strongly associated with poor patient prognosis and can increase the risk of death by 82% (11). Sepsis is considered one of the most common causes of perioperative death after percutaneous nephrolithotomy (12), with a mortality rate of 20-42% (13), and SIRS is the first step in the sepsis cascade and is closely associated with the development of sepsis (14), so systemic inflammatory response syndrome can be used as a sentinel for sepsis. Therefore, early recognition and timely intervention of systemic inflammatory response syndrome is the key to reduce the incidence of sepsis and patient mortality after PCNL. In recent years, there have been numerous studies on biomarkers to predict the occurrence of SIRS after PCNL, so this paper summarizes these biomarkers to predict the occurrence of systemic inflammatory response syndrome early after percutaneous nephrolithotomy.

## Procalcitonin

Procalcitonin is a protein consisting of 116 amino acids and is the peptide precursor of calcitonin (15). In normal physiological conditions, PCT is synthesized mainly in thyroid C cells and to a lesser extent by neuroendocrine tissues in other organs such as the lung and gastrointestinal tract (16). In response to stimulation induced by glucocorticoids, calcitonin gene-related peptides, glucagon, gastrin or β-adrenergic signals, PCT is converted to calcitonin before entering the circulatory system and therefore exhibits very low serum PCT levels (< 0.02 ng/mL) under normal physiological conditions (17). In contrast, during inflammation, PCT is mainly produced by two alternative mechanisms: a direct pathway induced by lipopolysaccharide (LPS) or other toxic metabolites from microorganisms and an indirect pathway induced by inflammatory mediators such as interleukin-6 (IL-6) and tumor necrosis factor-α (TNF-α) (18). Due to the lack of conversion of PCT to calcitonin under both alternative mechanisms, PCT enters the circulatory system directly, resulting in elevated PCT concentrations in the peripheral circulation.

PCT, as a biomarker most widely used in sepsis, is a good predictor of the occurrence, treatment outcome and prognosis of sepsis after PCNL (19, 20). Zheng et al. (19) found that when PCT > 0.3ng/ml its sensitivity to predict post-PCNL sepsis reached 90.3% and its specificity 94.3%. As similarly, the study (13) showed that PCT and C-reactive protein (CRP) were independent risk factors for SIRS after PCNL with good predictive effect and that 88.2% of patients with SIRS occurred within 24 hours after surgery. Gao et al. (21) showed that PCT at 2 hours postoperatively was more effective than CRP and white blood cell in predicting SIRS after PCNL, with a specificity of 87.6% and sensitivity of 75.4% for the diagnosis of SIRS when PCT > 3.7 ng/L at 2 hours postoperatively. This is analogous to the findings of Ding et al. (22). Therefore, PCT at 2 hours postoperatively is a biomarker to predict the occurrence of SIRS after PCNL. However, the sensitivity of PCT as a single indicator to predict postoperative SIRS is not high, and it needs to be combined with other indicators such as CRP to improve the sensitivity (13) for more accurate and sensitive identification of postoperative SIRS. In addition, the severity of postoperative infection can be well evaluated by dynamic monitoring of PCT (22) to guide treatment and thus prevent the misuse of antibiotics leading to antibiotic resistance.

## C-reactive protein

C-reactive protein is a biomarker of inflammation in the acute response phase stimulated by inflammatory factors such as interleukin-1 (IL-1), IL-6 and tumor necrosis factor (TNF) produced in the liver (23). CRP is currently widely used mainly in cardiovascular diseases (24), autoimmune diseases (25), oncology (26), and systemic infections (27), while fewer studies have been performed to predict the occurrence of SIRS after PCNL. Vishnu et al. (28) found that preoperative CRP was an independent risk factor for the occurrence of SIRS after PCNL based on a multifactorial logistic analysis, and by constructing a receiver operating characteristic curve (ROC) curve, they found that the preoperative CRP predicted the occurrence of SIRS after PCNL The best cut-off value for predicting SIRS after PCNL was 0.65 mg/dL, with a specificity of 69.4% and sensitivity of 51.4%, which was consistent with the study of Wang et al. (13). In addition, Wang et al. (13) showed that CRP can also predict the development of postoperative sepsis. However, it is not good in predicting SIRS or sepsis after PCNL compared to PCT (21), mainly because CRP is more susceptible to rheumatic diseases, malignancies and drug reactions compared to PCT (29). To address the limited role of CRP in predicting infection, a study found that the CRP-related index C reactive protein velocity (CRPv) (the difference between two CRP measurements before admission divided by the time between the two tests) can better distinguish sepsis from non-sepsis and has a better predictive value (23). However, there are no studies in which CRPv predicted the occurrence of SIRS after PCNL, so future randomized controlled studies with large samples should be conducted to verify the role of CRPv in predicting SIRS after PCNL.

## Interleukin-6

Interleukin-6 is a cytokine with multiple biological activities with pro- and anti-inflammatory activities, depending on the immune response environment. IL-6 is mainly produced by monocytes, neutrophils, T lymphocytes, B lymphocytes and NK cells and is involved in the development of systemic infections, autoimmune diseases and tumors through immune regulation (30, 31). In patients with kidney stones, bacteria are present not only in the urine but also in the stones, especially infected stones, and these bacteria and endotoxins are often released during PCNL lithotripsy. In addition, the high pelvic pressure caused by the flushing fluid leads to the entry of bacteria and endotoxins into the circulatory system through the damaged pelvic mucosa (32), which eventually leads to postoperative systemic severe reaction syndrome and even urogenic sepsis. Qi et al. (33) showed that compared to PCT, IL-6 at 2 hours postoperatively was the earliest and most valuable inflammatory biomarker for the diagnosis of urogenic sepsis occurring after PCNL, with an area under the ROC curve of 1.0. Unfortunately, however, their study did not provide an optimal cut-off value for IL-6 at 2 hours postoperatively to further guide the clinic. Similarly, Tang et al. (34) found that with compared to PCT and CRP, IL-6 at 12 hours postoperatively was the best for diagnosing urogenic sepsis after PCNL with a best cut-off value of 146.79 pg/mL, a specificity of 78.13% and a sensitivity of 73.44%. Therefore, IL-6 at 2 hours postoperatively is the earliest and most valuable biomarker of inflammation, but the diagnostic value of either PCT, CRP or IL-6 as a single indicator to predict the occurrence of infection after PCNL is limited and not as accurate as the combined diagnosis of the three indicators.

## Neutrophil CD64

CD64 is present on the surface of neutrophils and is a high-affinity receptor for the Fc portion of IgG. Under normal conditions CD64 is expressed at low levels on the surface of peripheral blood neutrophils. However, when the organism is in an infected state, the body produces large amounts of cytokines such as interferon-γ, IL-6, TNF-α, and granulocyte colony-stimulating factor, and these cytokines stimulate neutrophils to express CD64 in large amounts, and their expression peaks within 4 to 6 hours and remains stable for a certain period of time until 7 days after these cytokines return to normal and return to basal expression (35). Cong et al. (17) compared the value of CD64, PCT and IL-6 in the diagnosis of sepsis by Meta-analysis and found that CD64 had the highest diagnostic value for sepsis with a specificity of 88%, a sensitivity of 88% and an area under the ROC curve of 0.94. Given its stability and high diagnostic value for sepsis, CD64 can be used as a biomarker for predicting infection. There are not many studies of CD64 in predicting the emergence of SIRS after lithotripsy, focusing almost exclusively on the emergence of SIRS after ureteroscopic lithotripsy. A retrospective study based on 407 patients found that CD64 had the highest diagnostic value for the development of SIRS after ureteroscopy holmium laser lithotripsy compared to PCT versus CRP, with an area under the ROC curve of 1.0 at 2 hours, 6 hours, and 1 day postoperatively (36), similar to the findings of Fang et al. (37). Consequently, CD64 at 2 and 6 hours postoperatively is an effective biomarker for early prediction of SIRS after lithotripsy, but its validity in early prediction of SIRS after PCNL still needs further validation. In addition, there is no uniform unit of CD64 flow cytometry, and most of the different measurement units are not interconvertible, making it more difficult to apply the best cutoff value of CD64 for predicting the development of SIRS or sepsis.

## Monocyte HLA-DR

HLA-DR is a class II antigen and also a glycosylated transmembrane protein expressed on antigen-presenting cells. HLA-DR expressed on monocytes presents ingested pathogenic microbial peptides to CD4 or CD8-positive T cells, thereby initiating a specific immune response to eliminate the underlying pathogen. More studies have shown that reduced HLA-DR expression is a reliable diagnostic and prognostic biomarker for immunosuppression or sepsis in critically ill patients (38, 39). Patients with HLA-DR expression below 30% have a lower survival rate and a 30-fold increased risk of death compared to normal levels (40). Wu et al. (36) found that HLA-DR at 2 hours postoperative and 6 hours postoperative was a good predictor of SIRS after ureteroscopy holmium laser lithotripsy compared with the traditional inflammatory index PCT, but was less effective than CD64, which had significant areas of 0.993 and 0.983 for the 2-hour and 6-hour ROC curves, respectively. Considering the different surgical procedures, the resulting postoperative reaction stress is not the same. However, Hou et al. (41) found that HLA-DR could also be used to predict the development of sepsis after PCNL with an optimal cut-off value of 56.19%, specificity of 81.8% and sensitivity of 89.7%. Nevertheless, although SIRS and sepsis are different stages of development of the same disease, there are differences between them, mainly in organ dysfunction. Monocyte HLA-DR expression was found to be significantly lower in sepsis patients who developed acute renal failure compared to sepsis patients who did not (42). Therefore, the findings of Hou et al. (41) are not well applied in predicting the occurrence of SIRS after PCNL, and future clinical trials with large samples are still needed to validate the role of HLA-DR in predicting the occurrence of SIRS after PCNL.

## Complex inflammatory indicators

Blood tests are the most commonly used tests in clinical practice and are quick, economical and easy to perform, so they are often used to make preliminary judgments about inflammation in the body. For a long time, however, clinicians often choose white blood cell count, neutrophil count and neutrophil percentage to determine systemic inflammation, and rarely use other indicators of blood routine to judge, such as monocytes and lymphocytes. It was found that leukocytes are not as valuable in predicting or diagnosing infections compared to other indicators such as PCT and neutrophil-lymphocyte ratio (NLR) (19, 43), probably because some infections do not cause elevated or decreased leukocytes (44). For this reason, more and more studies are being conducted to improve the diagnostic value of systemic reactive syndrome or sepsis by combining the indicators from routine blood and liver function tests. Current composite inflammatory indicators used to predict or diagnose the development of SIRS after PCNL include neutrophil-lymphocyte ratio, lymphocyte-monocyte ratio (LMR), platelet-lymphocyte ratio (PLR), systemic systemic immune inflammation index (SII), prognostic nutritional index (PNI), albumin-globulin ratio (AGR), and hypersensitive C-reactive protein-albumin ratio (hs-CRP/Alb).

## Neutrophil-lymphocyte ratio

The neutrophil-lymphocyte ratio is now a common composite inflammatory index, originally described by Goodman et al. (45) in a 1995 study that evaluated its role in the diagnosis of appendicitis. Subsequently, as the relationship between tumors and inflammation was uncovered, more and more studies found that NLR was closely associated with the diagnosis and prognosis of urologic tumors such as prostate cancer (46), renal cell carcinoma (47), and adrenal cortical carcinoma (48). And similarly, NLR was strongly associated with predicting the occurrence of infection after minimally invasive treatment of urinary stones. The presence of kidney stones leads to the release of inflammatory mediators such as IL-6, IL-7, IL-8 and TNF-α, which in turn leads to an increase in neutrophil counts, while the inflammatory response suppresses the immune response by decreasing the cytolytic activity of lymphocytes, T cells and natural killer cells (12). Therefore, increased NLR may indicate a persistent inflammatory response. Sen et al. (49) found that NLR better predicted the development of sepsis after PCNL compared to white blood cell count with an optimal cut-off value of 2.5. This is in agreement with the findings of Kriplani et al. (12), in addition they also predicted the development of SIRS after PCNL by NLR with an optimal cut-off value of 2.03. However, NLR has a higher sensitivity and better value in predicting sepsis compared to predicting SIRS. Wang et al. (50) revealed that NLR was more effective in predicting the occurrence of SIRS after PCNL compared with the traditional index PCT, and its area under the ROC curve was higher than that of PCT with a specificity of 97%. In conclusion, NLR is a simple and easily available marker for predicting the occurrence of SIRS or sepsis after PCNL. In addition, NLR is a simple and easily available marker to predict the development of SIRS or sepsis after PCNL compared to more expensive tests such as PCT and IL-6, but the lack of consensus on the optimal cut-off value for NLR limits its use in clinical practice, and therefore prospective multicenter studies with large samples are needed to improve evidence and standardization.

## Lymphocyte-monocyte ratio

As with NLR, the lymphocyte-monocyte ratio is a commonly used indicator of compound inflammation. Several studies have shown the value of LMR in the diagnosis and prognostic assessment of various diseases such as malignancies and SIRS (51, 52). Winkler et al. (38) observed an increase in the number of monocytes during sepsis, while SIRS or sepsis decreased the circulating blood lymphocyte count. Thus, a lower LMR may reflect the inflammatory state. LMR predicted the occurrence of SIRS after PCNL was originally described in a study by Tang et al. (53), but its predictive power was not as good as NLR. This is in agreement with the findings of Kriplani et al. (12), who calculated the best cut-off value of 3.23, sensitivity of 83.9%, specificity of 42% and area under the curve of 0.649 for LMR prediction of SIRS by ROC curve, but they found that LMR predicted postoperative sepsis better than NLR with a best cut-off value of 2.88, sensitivity of 87.5%, specificity of 55%, and area under the curve of 0.726. It is evident that LMR is a better predictor of severe infection, and they found that LMR is an independent risk factor for the development of SIRS after PCNL by multifactorial logistic regression analysis. This is in agreement with the study of Xu et al. (54), whose optimal cut-off value was 3.4. On the flipside, the best cut-off values from a large number of relevant studies were smaller than the mean value of LMR in a healthy Chinese population (male: 5.14; female: 5.50) (12, 53, 55). Therefore, LMR is a biomarker that can predict SIRS or sepsis. Therefore, LMR is a biomarker that can predict SIRS or sepsis, but its specificity is low and needs to be combined with other indicators to further improve the diagnostic efficacy which means reducing the misdiagnosis rate.

## Platelet-lymphocyte ratio

The platelet-lymphocyte ratio is a novel composite inflammatory marker in recent years and has been shown to be predictive of a variety of diseases, such as diabetic complications and cancer (56, 57). Numerous current studies have shown that platelets are involved in the pathophysiological processes of sepsis and play a key role in organ dysfunction (58); while lymphopenia is a common marker of sepsis-induced immunosuppression (59). Therefore, PLR may be a biomarker for predicting systemic infection. Kriplani et al. (12) conducted a retrospective analysis of 517 post-PCNL patients and found that preoperative PLR was an independent risk factor for the development of SIRS after PCNL, with a specificity of 50.5% and sensitivity of 80.2% for predicting SIRS when the preoperative PLR was >110.62, which was less sensitive than NLR and LMR. This is similar to the findings of Cetinkaya et al. (14), who concluded that patients’ vital signs should be closely monitored and alerted for the development of postoperative SIRS when the preoperative PLR is >114.1. However, Tang et al. (53) found through a retrospective study that although PLR was statistically different between the non-SIRS and SIRS groups, PLR was not an independent risk factor for predicting the occurrence of SIRS after PCNL by multifactorial logistic analysis, which may be caused by the inherent limitations of the type of retrospective study, so a prospective study with a large sample should be conducted in the future to further verify its validity.

## Systemic immune inflammatory index

The systemic immune inflammatory index was first proposed by Hu et al. as a novel index of inflammation obtained by platelet * NLR calculation (60). Currently, SII is mainly applied in the prognosis of cardiovascular diseases and tumors (61, 62), and there are fewer studies in predicting postoperative complications, especially in SIRS or sepsis. There is only one national and international study on SII predicting the occurrence of SIRS after PCNL (9). Their retrospective analysis of 365 patients revealed that SII was an independent risk factor for the development of SIRS after PCNL and had a greater predictive value compared to NLR, LMR and PLR with a sensitivity of 79.63% and a specificity of 73.93%. This is probably because these predictors become unstable when only one or two parameters are involved and are usually susceptible to other confounding factors (63). In contrast, SII contains three parameters that are more stable and more objective in reflecting the balance between host inflammation and immune status (64). Therefore, SII is expected to be a biological indicator to predict the occurrence of SIRS after PCNL surgery. However, their study lacked the optimal cut-off value to directly guide the clinic, so the specific application of SII in the clinic, such as predicting diagnosis and guiding treatment, should be further investigated in the future.

## Prognostic nutritional index

The prognostic nutritional index was first proposed by Onodera et al. in 1984 and was used to assess the risk of surgery for gastrointestinal tumors to guide treatment and prognosis with good results (65). Studies after this have focused on tumor prognosis, such as Li et al. who found that PNI >50.2 had a more favorable prognosis for prostate cancer (66). PNI is calculated based on serum albumin level and peripheral lymphocyte count, i.e. serum albumin concentration (g/L) + 5 * total peripheral circulating lymphocyte count (10^9^/L), which can reflect the nutritional, inflammatory and immune status of the patient in a simpler and more comprehensive way. Nutrition affects all physiological processes, including those related to the development and function of our immune system (67), so malnutrition often leads to immune dysfunction (68), which in turn leads to a greater susceptibility of patients to postoperative infections. There are few studies involving PNI to predict the occurrence of SIRS after PCNL. Xu et al. (54) found that PNI was an independent influencing factor for the occurrence of SIRS after PCNL through a retrospective analysis of 556 patients, and the probability of SIRS could be reduced when PNI was >49. They also found that PNI predictive value was greater than PLR and comparable to NLR and LMR. In addition, PNI is significantly more effective than NLR and PLR in predicting all-cause mortality in sepsis (69), so early nutritional support for patients with sepsis may reduce mortality in patients (70). Consequently, PNI may be a potential inflammatory marker for predicting the occurrence of SIRS after PCNL, but future clinical trials with large samples as well as exploring the relationship between it and SIRS should still be conducted to provide a basis for the future application of PNI in the clinic.

## Albumin-globulin ratio

Albumin and globulin are important components of serum proteins and play a potential role in the systemic inflammatory response. Low clear albumin concentrations not only reflect malnutrition but also predict infectious complications after oncologic surgery and orthopedic surgery (71, 72). Globulins are acute phase proteins during the host immune response and their concentrations increase shortly after the invasion of pathogens and toxins into the body, and high concentrations indicate a state of systemic inflammation and accumulation of various inflammatory cytokines (73). Therefore, the albumin-globulin ratio, like the PNI, provides a simpler and more comprehensive picture of the patient’s nutritional, inflammatory and immune status. Xun et al. (74) showed that AGR was an independent risk factor for postoperative sepsis in PCNL by multifactorial logistic analysis, and they found that the incidence of sepsis decreased with increasing AGR, and the incidence of postoperative sepsis was 0.78% when AGR was ≥1.5; 5.93% when AGR was between 1 and 1.5; 11.59% when AGR <1.0, the incidence of postoperative sepsis was 11.59%. Therefore, preoperative AGR should be kept above 1.5 to greatly reduce the incidence of sepsis. Wang et al. (75) showed that AGR was also an independent risk factor for SIRS after PCNL, and they found that AGR had the best predictive efficacy compared to traditional inflammatory indicators such as neutrophils, total leukocytes, C-reactive protein, or a single indicator of albumin and globulin, with an optimal cut-off value of 1.145, at which point the sensitivity was 83.3% and the specificity was 88.9%. Therefore, it is recommended that patients with AGR <1.145 be carefully evaluated and treated before undergoing PCNL to reduce the occurrence of systemic inflammatory response syndrome after surgery. However, future multicenter clinical studies with large samples are still needed to determine the optimal cut-off value and influencing factors of AGR for better application in the clinic.

## Ultrasensitive C-reactive protein-albumin ratio

Ultrasensitive C-reactive protein-albumin is a novel inflammatory marker, in which ultrasensitive C-reactive protein is the same protein as C-reactive protein, which is produced by mainly hepatocytes and regulated by cytokines such as IL-1β, IL-6, and TNF, the only difference is that ultrasensitive C-reactive protein is detected in a more sensitive manner and can detect very low concentrations of CRP in plasma (76), so it can detect inflammatory responses earlier than CRP. Liao et al. (77) showed that when hs-CRP/Alb > 0.102, it has a good predictive value for the development of SIRS after PCNL with a sensitivity of 94.8% and a specificity of 75.5%. This is consistent with the study of Xu et al. (54), who showed by ROC curve analysis that hs-CRP/Alb has better predictive value than NLR, PLR, LMR and PNI for postoperative SIRS with an optimal cut-off value of 0.06, sensitivity of 76.4% and specificity of 73.2%. They found that an elevated hs-CRP/Alb ratio was associated with female gender, preoperative urine culture, hs-CRP, albumin, hemoglobin, and creatinine. Therefore, the influence of these factors on hs-CRP/Alb should be further explored in the future when hs-CRP/Alb is used in actual clinical practice.

## Conclusions

Sepsis is the most serious complication after percutaneous nephrolithotomy and is one of the leading causes of death after PCNL, of which more than half progress from systemic inflammatory response syndrome, so SIRS can be used as a sentinel for sepsis. There is growing evidence that biomarkers such as traditional inflammatory markers (PCT, CRP and IL-6), novel inflammatory markers (neutrophil CD64 and monocyte HLA-DR), and composite inflammatory markers (NLR, LMR, PLR, SII, PNI, AGR and hs-CRP/Alb) play an important role in early prediction of postoperative development of SIRS (**Table 1**). However, it is worth noting that the novel inflammatory indicators are currently focused on studies of the development of SIRS after ureteroscopic lithotripsy, so their effectiveness in early prediction of the development of SIRS after PCNL will remain to be further validated in the future. In addition, the cut-off value of each biomarker varies somewhat in different studies, which limits its application in clinical practice. Therefore, in the future, it is still necessary to determine the optimal cut-off value of these biomarkers through prospective studies or Meta-analysis with large samples and multiple centers; secondly, the role of a single indicator is limited, and in the future, these indicators should be combined to improve the sensitivity and specificity of prediction in order to better guide timely clinical detection of postoperative SIRS and early intervention to reduce the incidence of postoperative sepsis and patient mortality.

**Table 1:** Biomarkers.

Biomarkers	PCNL/URL	Sepsis/SIRS	Cut-off	Sensitivity (%)	Specificity(%)	OR	AUC	Use time	References
PCT	PCNL and URL	SIRS	-	-	-	1.093(1.005-1.187)	-	Within 24h after surgery	Wang et al. (13)
	PCNL and URL	Sepsis	-	-	-	1.017(1.006-1.029)	-	Within 24h after surgery	Wang et al. (13)
	PCNL	Sepsis	0.3ng/ml	90.3	94.3	-	0.960	Postoperative	Zheng et al. (19)
	PCNL	SIRS	3.7ng/L	75.4	87.6	-	0.852	2h postoperative	Gao et al. (21)
	PCNL	SIRS	3.5ng/L	-	-	-	0.694	Postoperative	Ding et al. (22)
CRP	PCNL and URL	SIRS	-	-	-	1.017(1.009-1.024)	-	Within 24h after surgery	Wang et al. (13)
	PCNL and URL	Sepsis	-	-	-	1.080(1.042-1.120)	-	Within 24h after surgery	Wang et al. (13)
	PCNL	SIRS	0.65 mg/dL	51.4	69.4	1.59 (1.07-2.37)	0.63	Preoperative	Vishnu et al. (28)
IL-6	PCNL	Sepsis	-	-	-	-	1.000	2h postoperative	Qi et al. (33)
	PCNL	Sepsis	146.79 pg/mL	73.44	78.13	-	0.856	12h postoperative	Tang et al. (34)
nCD64	URL	SIRS	-	-	-	-	1.000	6h postoperative	Wu et al. (36)
	URL	SIRS	-	-	-	-	0.999	6h postoperative	Fang et al. (37)
mHLA-DR	URL	SIRS	-	-	-	-	1.000	6h postoperative	Wu et al. (36)
	PCNL	Sepsis	56.19%	89.7	81.8	-	0.934	1d postoperative	Hou et al. (41)
NLR	PCNL	SIRS	2.03	82	31	-	0.596	Postoperative	Kriplani et al. (12)
	PCNL	Sepsis	2.45	87	31	-	0.639	Postoperative	Kriplani et al. (12)
	PCNL	Sepsis	2.5	-	-	-	0.558	Preoperative	Sen et al. (49)
	PCNL	SIRS	3.49	54.1	97	4.336(1.630-11.534)	0.807	Preoperative	Wang et al. (50)
LMR	PCNL	SIRS	3.23	83.9	42	-	0.831	Postoperative	Kriplani et al. (12)
	PCNL	Sepsis	2.88	87.5	55	-	0.726	Postoperative	Kriplani et al. (12)
	PCNL	SIRS	-	-	-	-	0.723	Postoperative	Tang et al. (53)
	PCNL	SIRS	3.4	-	-	-	0.633	Preoperative	Xu et al. (54)
PLR	PCNL	SIRS	110.62	80.2	50.5	-	0.663	Postoperative	Kriplani et al. (12)
	PCNL	Sepsis	120.25	87.5	53.2	-	0.627	Postoperative	Kriplani et al. (12)
	PCNL	SIRS	114.1	80.4	60.2	1.01(1.002-1.022)	0.731	Preoperative	Cetinkaya et al. (14)
	PCNL	SIRS	-	-	-	-	0.685	Postoperative	Tang et al. (53)
SII	PCNL	SIRS	480.37	79.63	73.93	2.951(1.370-6.355)	0.786	Preoperative	Peng et al. (9)
PNI	PCNL	SIRS	49	-	-	0.559(0.338-0.924)	0.629	Preoperative	Xu et al. (54)
AGR	PCNL	Sepsis	1.5	-	-	5.068(1.135-22.624)	0.650	Preoperative	Yang et al. (74)
	PCNL	SIRS	1.145	83.3	88.9	0.048(0.010-0.239)	0.844	Preoperative	Wang et al. (75)
hs-CRP/Alb	PCNL	SIRS	0.06	76.4	73.2	6.925(4.244-11.300)	0.791	Preoperative	Xu et al. (54)
	PCNL	SIRS	0.102	94.5	75.5	-	0.934	Preoperative	Liao et al. (77)

PCT, Procalcitonin; CRP, C-reactive protein; IL-6, Interleukin-6; nCD64, Neutrophil CD64; mHLA-DR, Monocyte HLA-DR; NLR, neutrophil-lymphocyte ratio; LMR, lymphocyte-monocyte ratio; PLR, platelet-lymphocyte ratio; SII, systemic systemic immune inflammation index; PNI, prognostic nutritional index; AGR, albumin-globulin ratio; hs-CRP/Alb, hypersensitive C-reactive protein-albumin ratio; PCNL, percutaneous nephrolithotomy; URL, Ureteroscope Lithotripsy; SIRS, systemic inflammatory response syndrome; OR, odd ratio. -, Unavailable.

## Author contributions

WW: Conceptualization, literature search, and writing the article. DZ: Writing the article. TJ, TL and FZ: Reviewing the article. All authors contributed to the article and approved the submitted version.
